# Molecular Mechanisms of Mouse Skin Tumor Promotion

**DOI:** 10.3390/cancers2020436

**Published:** 2010-04-13

**Authors:** Joyce E. Rundhaug, Susan M. Fischer

**Affiliations:** The University of Texas M. D. Anderson Cancer Center, Science Park–Research Division, P.O. Box 389, Smithville, TX 78957, USA; E-Mail: jrundhaug@mdanderson.org (J.E.R.)

**Keywords:** skin carcinogenesis, tumor promoters, 12-*O*-tetradecanoylphorbol 13-acetate (TPA), protein kinase C (PKC), epidermal growth factor receptor (EGFR), transforming growth factor-β (TGFβ), tumor necrosis factor-α (TNFα), interleukins, cyclooxygenase-2 (COX-2), ornithine decarboxylase (ODC)

## Abstract

Multiple molecular mechanisms are involved in the promotion of skin carcinogenesis. Induction of sustained proliferation and epidermal hyperplasia by direct activation of mitotic signaling pathways or indirectly in response to chronic wounding and/or inflammation, or due to a block in terminal differentiation or resistance to apoptosis is necessary to allow clonal expansion of initiated cells with DNA mutations to form skin tumors. The mitotic pathways include activation of epidermal growth factor receptor and Ras/Raf/mitogen-activated protein kinase signaling. Chronic inflammation results in inflammatory cell secretion of growth factors and cytokines such as tumor necrosis factor-α and interleukins, as well as production of reactive oxygen species, all of which can stimulate proliferation. Persistent activation of these pathways leads to tumor promotion.

## 1. Introduction

The development of cancer is a complex process during which a normal cell undergoes a progressive series of alterations resulting in the acquisition of an altered proliferative capacity, invasiveness and metastatic potential. These alterations are classically defined as occurring in stages: initiation involves DNA damage leading to mutation(s); this is followed by promotion, which involves enhanced proliferation and altered cell behavior; and finally progression results from subsequent genetic changes such as loss of heterozygosity and gene amplification. Classical tumor promoters are not mutagenic like carcinogens but rather cause an alteration of the expression of genes whose products are associated with hyperproliferation, tissue remodeling and inflammation. Ultraviolet (UV) radiation, on the other hand, is both mutagenic and tumor promoting and can therefore cause initiation as well as promote the clonal expansion of initiated cells.

**Figure 1:**
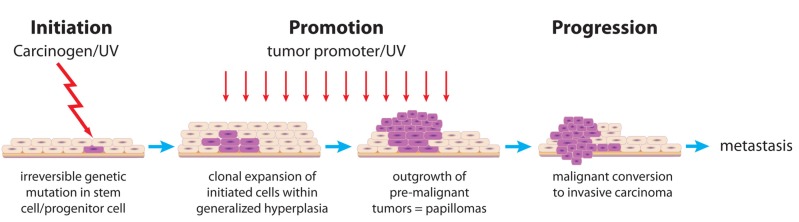
Multistage mouse skin carcinogenesis model.

The mouse skin model of multistage carcinogenesis (see Figure 1) has been extensively used to study molecular changes associated with the dysregulated signaling and altered gene expression relevant to the various stages of tumor development. Thus, this review on the mechanisms of tumor promotion focuses on this model system, although most of the principles are relevant to other epithelial tissues. The effects of different tumor promoters, as well as modulation of tumor promotion in various transgenic and knockout mice, in the skin model have illuminated the molecular mechanisms involved in promotion of tumorigenesis.

Initiation occurs by exposure of the skin to either a chemical carcinogen or ultraviolet (UV) light, which causes a genetic mutation (either an activating mutation in a proto-oncogene such as H-*ras* or an inactivating mutation in a tumor suppressor gene such as *p53*) in the stem cell or progenitor cell compartment. Repeated exposure of the skin to chemical tumor promoters, UV light or other irritants including wounding promotes the clonal expansion of the initiated cell(s) as well as a generalized hyperplasia of the epidermis. Eventually initiated cells outgrow the normal epidermal cells to form premalignant papillomas. With further genetic changes, some of the papillomas convert to malignant carcinomas capable of invading through the basement membrane and finally metastasizing to the lymph nodes and lungs.

One of the cellular processes that is crucial for skin tumor promotion is the induction of cell proliferation and maintenance of a sustained hyperplasia [1]. Initiated cells respond differently to repeated mitogenic stimuli and this allows their clonal expansion beyond that of the surrounding hyperplastic, but otherwise normal epidermis. Regenerative proliferation, such as that due to repeated wounding or ultraviolet (UV) light exposure, can promote tumorigenesis as well as chemicals and endogenous growth factors that induce cell proliferation [1]. Alternatively, stimulation of cell signaling pathways that promote cell survival and/or inhibit apoptosis can be tumor promoting [2].

Another common feature of tumor promotion is induction of chronic inflammation. Tumor promoter-induced secretion of pro-inflammatory molecules by keratinocytes results in the recruitment of inflammatory cells, e.g., leukocytes, lymphocytes and macrophages, into the dermis. These activated cells then produce growth factors, cytokines and chemokines that promote cell proliferation, matrix remodeling, angiogenesis and suppression of adaptive immunity, all of which promote tumor growth [3,4,5]. In addition, activated inflammatory cells produce reactive oxygen species (ROS) and nitric oxide resulting in oxidative stress, which has been shown to be associated with tumor promotion [4,6,7,8]. For example, topical application of the contact allergen 2,4-dinitrofluorobenzene elicits a strong dermal inflammatory response and repeated treatments promote skin tumorigenesis [9].

ROS can also be produced in the target cells directly by tumor promoters. UVA wavelengths, which penetrate deeply into the skin, induce formation of ROS and oxidative stress in both epidermal keratinocytes and dermal fibroblasts [7,10,11]. The classical skin tumor promoter, 12-*O*-tetradecanoylphorbol-13-acetate (TPA), has been shown to increase production of ROS and hydroperoxides in keratinocytes both *in vitro* and *in vivo* [12,13] and organic peroxides have been shown to promote skin tumorigenesis [14,15].

In general, the effects of tumor promoters are reversible for a limited number of applications; however, their prolonged epigenetic effects result in irreversible genetic events in the later stages of tumor promotion [1]. So what are the molecular mechanisms by which tumor promoters cause changes in cell proliferation and gene expression? Tumor promoters, whether UV, chemicals or endogenous factors, usually interact at the cell’s surface with specific receptors or other cell components that elicit several processes/responses, including enhanced DNA synthesis, increased production of eicosanoids, cytokines and growth factors, a pro-oxidant state and alterations in cell surface properties leading to changes in cell adhesion and cell-cell communication. Tumor promotion leads to altered gene expression and identification of these critical events offers targets for chemoprevention and/or therapy.

## 2. Receptors for Tumor Promoters

### 2.1. Protein Kinase C (PKC)

Early mouse skin carcinogenesis studies were performed using a low dose of a carcinogen, such as 7,12-dimethylbenz[*a*]anthracene (DMBA) or benzo[*a*]pyene (B[*a*]P), followed by repeated applications of an irritant such as croton oil (see e.g., [16]). Later, the active components of croton oil were identified as phorbol esters with TPA being the most potent. Finally, in the early 1980s, the receptor for phorbol esters was identified as PKC, which is a calcium-activated, phospholipid-dependent serine/threonine kinase [17]. Diacylglycerol (DAG) is the endogenous ligand for PKC. DAG is generated along with inositol 1,4,5-trisphosphate by cleavage of phosphatidylinositol 4,5-bisphosphate by phospholipase C (PLC) [18,19]. Multiple isoforms of PKC have since been identified, some of which are not calcium- or DAG-activated. Several other non-phorbol ester classes of tumor promoters, despite apparent structural differences, have also been shown to bind to and activate PKC, including mezerein, teleocidins, lyngbyatoxin and aplysiatoxins [20,21].

#### 2.1.1. Structure and Isozymes

Nine PKC isozymes have been identified and they are classified into three groups based on their structure and cofactor requirements. All of the isozymes have a N-terminal regulatory region containing a short pseudosubstrate domain that inhibits enzyme activity immediately preceding one or two cysteine-rich conserved C1 domains that form the phorbol ester/DAG binding site [22,23]. A variable hinge region separates the regulatory region from the C-terminal catalytic region containing the C3 ATP-binding domain and C4 substrate-binding domain [22,23]. The first discovered and best characterized group is the conventional or classical PKCs, which include PKCα, PKCβ and PKCγ. These isozymes also contain a C2 domain in the regulatory region that binds acidic lipids such as phosphatidylserine and calcium, which are required cofactors for their activity [22,23]. The second group is the novel PKCs, which are calcium-independent and include PKCδ, PKCε, PKCη and PKCθ. The atypical PKCs, PKCζ and PKCλ (human homolog is called PKCι), contain only one C1 domain and are not activated by phorbol esters or DAG [19,22]. PKCα, PKCβ, PKCδ, PKCε, PKCζ and PKCη are the isoforms expressed in human and mouse skin [24,25,26,27,28].

#### 2.1.2. Activation and Substrates

Newly synthesized PKCs associate with the membrane where they are phosphorylated in the activation loop of the catalytic domain by 3-phosphoinositide-dependent kinase-1 (PDK-1) [29,30,31]. This phosphorylation is essential for their catalytic activity and leads to autophosphorylation of two sites in the C1 and C2 domains, which releases the enzyme into the cytosol where it remains inactive though interaction of the pseudosubstrate domain with the substrate-binding domain [30]. Upon displacement of the pseudosubstrate domain by ligands such as DAG, the enzyme becomes catalytically active [22,23]. After activation, the PKC isozymes are translocated from the cytosol to specific subcellular membrane sites where they interact with specific receptors of activated C-kinases (RACKs) [30]. Membrane localization allows interaction with phosphatidylserine or other acidic lipid cofactors and greatly increases the affinity of conventional PKCs for calcium, both of which are needed for full activity [22,23]. In addition, PKCδ is uniquely activated by caspase-3 cleavage in the hinge region generating a soluble (rather than a membrane-associated) catalytically active fragment, which in turn can active pro-caspase-3, thereby amplifying apoptotic signaling [32,33].

DAG is rapidly metabolized and thus activates PKC only transiently, while TPA persists much longer and results in prolonged activation of PKC [22,23,34]. However, sustained activation of conventional and novel PKCs leads to desensitization and down-regulation. Active PKC traffics to the endosomes *via* internalization of caveolae and is then sorted to a perinuclear compartment, where PKC is dephosphorylated rendering it inactive [35,36]. However, for PKCδ, phosphorylation is required for its subsequent degradation [37]. PKC is then ubiquitinated and degraded *via* the proteasome pathway [38,39]. So while TPA binding to PKC leads to its persistant activation, the prolonged activation also results in PKC down-regulation.

Numerous proteins have been identified as substrates for PKC (see [23] for a list of 110 proteins). Only a few of the substrates and downstream signaling pathways relevant to tumor promotion will be mentioned here. PKC isoforms have been shown to activate the Ras/Raf/mitogen-activated protein kinase (MAPK) cascade and to mediate growth factor-stimulated activation of MAPK/extracellular signal-regulated kinase (ERK) and cell proliferation [40,41,42]. Activation of this cascade by PKCs is complex and regulated at several levels. PKCα and PKCε have been shown to directly phosphorylate and activate c-Raf-1 [43,44]. However, others have shown that targeting of c-Raf-1 to the membrane *via* Ras may be the mechanism by which PKCα and η activates c-Raf-1, while phosphorylation by PKCα and βI may be involved in the desensitization of c-Raf-1 [40]. In addition, conventional and atypical PKCs have been shown to phosphorylate Raf kinase inhibitory protein (RKIP) causing its dissociation from Raf-1 leading to activation of the downstream MAPK/ERK pathway [45].

Another MAPK, c-Jun N-terminal kinase (JNK), which is preferentially activated by cellular stress and inflammatory cytokines, has also been shown to be activated by phorbol esters and PKC. PKC phosphorylation of JNK at Ser^129^ requires RACK1 and augments JNK activation by its upstream kinases MKK4 and MKK7 [46]. Ultraviolet (UV) light, which can promote as well as initiate skin carcinogenesis, rapidly and potently activates JNK specifically *via* PKCδ phosphorylation at Ser^129^ [47].

The epidermal growth factor receptor (EGFR) is directly phosphorylated by PKC on three threonine sites, in particular Thr^654^ in the cytoplasmic juxtamembrane region, and it was initially reported that this phosphorylation reduces EGFR tyrosine kinase activity [48,49,50]. However, subsequent studies using mutant forms of EGFR have shown that neither phosphorylation at the PKC site Thr^654^ nor at a MAPK kinase (MEK) site Thr^669^ are sufficient for TPA/PKC inhibition of ligand-stimulated EGFR tyrosine kinase activity [51,52]. It has since been demonstrated that while PKC phosphorylation of EGFR inhibits subsequent EGF-induced EGFR activation, pretreatment with EGF prevents the inhibitory effects of phorbol esters on EGFR signaling [53]. This study also suggested that PKC-mediated juxtamembrane phosphorylation of EGFR and its related family member ErbB2 transiently amplifies EGFR signaling by enhancing the stability of liganded receptor oligomers, but this also enhances the internalization of the receptors [53]. The latter effect explains the long-known phenomenon of loss of high affinity EGFR binding sites after TPA treatment or PKC phosphorylation of EGFR [54,55,56].

Additional PKC substrates that contribute to crosstalk with other signaling pathways include guanylate cyclase [57] and adenylate cyclase [58] with PKC phosphorylation resulting in enhanced activity for both. On the other hand, PKCα phosphorylation of the catalytic subunit of phosphatidylinositol-3-kinase (PI3K) decreases its lipid kinase activity [59]. Since PI3K activity leads to PDK-1 activation and PDK-1 phosphorylates and activates PKC, this may represent a negative feedback loop to limit PI3K signaling. PKCζ and PKCα activate the nuclear factor-κB (NF-κB) signaling pathway by phosphorylating and activating IκB kinase β (IKKβ), which in turn phosphorylates and targets for degradation IκB [60]. However, at least in colon cancer cells, prolonged activation of PKC by TPA inhibits NF-κB signaling by disrupting the IKKγ/heat shock protein 90 (Hsp90) complex leading to proteasome degradation of IKKγ and subsequently IKKβ [61].

Other recently identified substrates of PKCs include vimentin and RASSF1A. Phosphorylation of vimentin, an intermediate filament upregulated upon epithelial cell transformation, by PKCε promotes integrin recycling and cell motility [62]. RASSF1A (Ras association domain family 1A) is a tumor suppressor and is phosphorylated by PKC at Ser^197^ and Ser^203^ in the Ras association domain, which blocks its ability to reorganize the microtubule network allowing cell cycle progression [63].

#### 2.1.3. Roles of PKCs in Tumor Promotion

Altered expression of PKC isozymes has been observed in a number of human cancers. While most studies report down-regulated expression of PKCα, β and δ in human cancers, PKCε has often been found to be overexpressed in various cancers relative to the normal tissue [19]. Similarly for mouse skin carcinogenesis, transgenic and knockout mouse models have demonstrated distinct roles for different PKC isoforms.

PKCα is the major conventional PKC isoform in the epidermis and is highly expressed [64]. Transgenic overexpression of PKCα in the basal layer of the skin *via* a keratin 5 (K5) promoter does not affect keratinocyte growth or differentiation, but a hyperinflammatory response results from a single application of TPA [65,66]. The PKCα-induced inflammation is mediated by up-regulated keratinocyte expression of CXC chemokine receptor 2 (CXCR2) ligands, which recruit infiltrating neutrophils [67]. Although overexpression of PKCα does not affect skin tumorigenesis in the standard DMBA/TPA protocol [65], when a low dose of TPA is used, tumor development and conversion to carcinomas is greatly enhanced in K5.PKCα transgenic mice [68]. On the other hand, PKCα null mice have increased susceptibility to skin carcinogenesis even though TPA-induced epidermal hyperplasia is reduced [69]. Thus, PKCα appears to be important for TPA induction of proliferation and inflammation, but it has contradictory roles in skin tumor promotion.

PKCδ is expressed in the basal cells of the epidermis and it contributes to the early onset of differentiation and keratinocyte apoptosis [19,64]. Transgenic mice that overexpress PKCδ in the basal layer *via* a keratin 14 (K14) promoter are resistant to standard two-stage skin tumorigenesis in that they develop significantly fewer number of papillomas and carcinomas [70]. Suppression of TPA tumor promotion by PKCδ overexpression results from enhanced TPA-induced apoptosis and inhibition of TPA-induced proliferation [71]. On the other hand, UV induction of skin tumors, apoptosis and epidermal proliferation are similar in K14.PKCδ transgenic mice as in wild-type mice [71]. While transgenic overexpression of PKCδ has no effect on UV-induced apoptosis, retroviral transduction of a caspase-resistant mutant PKCδ into human keratinocytes prevents apoptosis induced by UV [33]. These data clearly demonstrate the pro-apoptotic function of PKCδ activated by cleavage by caspase-3. Thus, although transgenic PKCδ does not affect UV tumorigenesis, it counteracts TPA tumor promotion.

PKCη is expressed primarily in the suprabasal granular layer of the epidermis and regulates cornified envelope formation [64]. Cholesterol sulfate, which activates PKCη, stimulates epidermal differentiation and suppresses skin carcinogenesis when applied before each tumor promoter treatment [72]. PKCη null mice show enhanced susceptibility to skin tumorigenesis, prolonged hyperplasia in response to TPA and delayed wound healing [73]. Together, these results suggest that PKCη inhibits tumor promotion through its regulation of epidermal differentiation and homeostasis.

PKCε is expressed in basal keratinocytes and its activation leads to proliferation and modulation of integrin-mediated cell adhesion [64]. K14.PKCε transgenic mice are phenotypically normal at birth, but as the mice age they develop chronic inflammation, hyperproliferation and/or ulceration around the tail base, ears and eyes [74]. Although these transgenic mice develop much fewer papillomas than wild-type mice in chemical and UV skin carcinogenesis protocols, more squamous cell carcinomas develop that appear *de novo* without going through a papilloma stage and that rapidly metastasize to regional lymph nodes [74,75,76]. The level of PKCε transgene expression correlates with epidermal expression of tumor necrosis factor-α (TNFα) and both TPA and UV induce greater TNFα expression and release of TNFα into the serum in transgenic compared to wild-type mice [76,77]. As described in more detail below, TNFα is a key tumor promotion cytokine that regulates many cellular responses including inflammation, immunity, cell proliferation, differentiation and apoptosis [78]. Indeed, PKCε transgenic mice are resistant to UV-induced caspase-8 activation and apoptosis [78]. In addition, PKCε overexpression enhances TPA- and UV-induced activation of signal transducer and activator of transcription-3 (Stat3) *via* direct interaction with and phosphorylation of Stat3 at Ser^727^ [79]. Stat3 activation has also been shown to be essential for skin tumor promotion [80,81]. Thus, PKCε promotes skin carcinogenesis *via* several mechanisms including among others, induction of TNFα and activation of Stat3.

In summary, tumor promoter activation of several PKC isoforms, in particular PKCα and PKCε, is critical for induction of proliferation and inflammation that is necessary for skin tumor development. Additionally, PKCε-mediated changes in cell adhesion and motility enhance malignant progression. Conversely, down-regulation of other PKC isoforms, such as PKCδ and PKCη, also contributes to tumor promotion in the skin by attenuating epidermal differentiation and apoptosis [64]. 

### 2.2. Aryl Hydrocarbon Receptor (AhR)

The AhR, a basic helix-loop-helix transcription factor, mediates the toxic and carcinogenic effects of polycyclic aromatic hydrocarbons (PAHs) and halogenated PAHs [82]. Unliganded AhR is sequestered in a cytoplasmic complex that includes Hsp90, hepatitis B virus X-associated protein 2 (XAP2), p23 and c-Src [83,84]. Upon ligand binding, AhR is released from its chaperone complex, translocates to the nucleus and heterodimerizes with a related family member, aryl hydrocarbon nuclear translocator (Arnt) [82]. AhR/Arnt then recruits co-activators and/or co-repressors in the nucleus and binds to xenobiotic response elements (XREs) to regulate the transcription of target genes [85]. The classical AhR target gene is cytochrome P450 *CYP1A1*, which is a member of the phase I monooxygenase enzyme superfamily that is involved in xenobiotic metabolism [84]. Bilirubin, lipoxin A4 and the tryptophan derivative 6-formylindolo[3,2-b]carbazole (FICZ) have been proposed to be endogenous ligands for AhR [85]. In some situations ligand-independent activation of AhR has been demonstrated [82,85]. After ligand binding and nuclear translocation, AhR is rapidly degraded, which limits its signaling [85].

AhR is expressed in the skin and its xenobiotic ligand 2,3,7,8-tetrachlorodibenzo-*p*-dioxin (TCDD) can promote skin tumorigenesis in HRS/J hairless (*hr/hr*), although not in haired (*hr*/+) mice [86]. PAH carcinogens bind to AhR and thereby induce cytochrome P450 enzymes, which are necessary for their metabolic activation. In complete skin carcinogenesis protocols, repeated applications of carcinogen induce tumor formation. Not surprisingly then, AhR null mice are completely resistant to B[*a*]P-induced complete skin carcinogenesis, as well as to B[*a*]P-induced subcutaneous tumorigenesis [87]. Transgenic mice that overexpress a constitutively active form of AhR in the skin *via* a K14 promoter develop inflammatory skin lesions with up-regulated expression of multiple inflammatory cytokines in the epidermis [88]. Recent reports have demonstrated that AhR plays a role in UV-induced responses in keratinocytes. Tryptophan absorption of UV irradiation leads to formation of FICZ, which is a high affinity ligand for AhR [89]. This results in nuclear translocation of AhR and induction of CYP1A1 expression. At the same time c-Src is released from the AhR/Hsp90 complex and activates EGFR/MAPK leading to induction of cyclooxygenase-2 (COX-2) [84,90]. UV induction of both CYP1A1 and COX-2 in the skin are completely blocked in AhR null mice [90].

Taken together, these data indicate that activation of AhR plays various roles in skin carcinogenesis by being the receptor for PAH carcinogens and inducing their activation, provoking inflammation in the skin and mediating some of the UV responses in the skin.

### 2.3. Peroxisome Proliferator-Activated Receptors (PPARs)

PPARs are in the nuclear hormone receptor superfamily and were identified in the 1990s as the receptors for peroxisome proliferators. PPARs form heterodimers with another superfamily member, the retinoid receptor RXR, and bind to peroxisome proliferator response elements (PPREs) in the promoters of target genes [91]. Three PPAR isoforms encoded by separate genes are differentially expressed: PPARα is expressed in the liver, kidney, intestine, heart, skeletal muscle, brown adipose tissue, adrenal gland, skin and pancreas; PPARβ (also called PPARδ) is ubiquitously expressed; and PPARγ is expressed primarily in adipose tissues and to a lesser extent in the intestine, retina, skeletal muscle and lymphoid tissues [91].

Endogenous ligands for PPARs include fatty acids and COX- and lipoxygenase (LOX)-generated eicosanoids. Long chain (C_16–20_) polyunsaturated fatty acids (e.g., linoleic, arachidonic and docosahexaenoic acids) bind to and activate all three isoforms of PPAR [92,93,94]. In a more isoform-specific manner, certain arachidonic acid metabolites are also effective ligands for the PPARs. Of LOX products, 8S-hydroxyeisocatetraenoic acid (8S-HETE), is a specific activator of PPARα [92,93,94], while 15S-HETE activates PPARγ [95]. Prostaglandins (PGs) derived from COX metabolism of arachidonic acid, PGA_1_ and PGD_2_, activate PPARβ/δ, while PGD_1_, PGD_2_ and 15-deoxy-∆^12,14^-PGJ_2_ activate PPARγ [92,94]. Thus, PPARs maintain lipid homeostasis by acting as sensors of fatty acid and lipid levels and then inducing the expression of fatty acid metabolizing enzymes.

In the skin tumorigenesis model, PPARα ligands conjugated linoleic acid and Wy-14,463 moderately inhibit TPA tumor promotion [96]. PPARα is found in the epidermis as well as in skin papillomas and carcinomas and is most highly expressed in cultured keratinocytes that have been induced to differentiate by high calcium levels [96]. The endogenous PPARα ligand 8S-HETE, which is produced by 8S-LOX after skin irritation, induces keratinocyte differentiation and PPARα-mediated transcription [97]. Like exogenous treatment with PPARα ligands, overexpression of 8S-LOX in the epidermis *via* a loricrin promoter, which results in elevated 8S-HETE levels, inhibits two-stage skin tumorigenesis [97,98]. Thus, in the skin, activation of PPARα inhibits tumor promotion.

The inflammatory cytokine, TNFα, induces PPARβ/δ expression in epidermal kertinocytes [99]. Cytokine-induced activation of PPARβ/δ enhances cell survival by up-regulating antiapoptotic and down-regulating proapoptotic genes, but also induces cell cycle arrest and terminal differentiation of keratinocytes [99]. Thus, PPARβ/δ may have either pro- or anti-tumorigenic activities depending on the context.

## 3. Growth Factors and Receptors

### 3.1. Epidermal Growth Factor Receptor (EGFR)/ErbB Family Signaling

#### 3.1.1. Receptors and Ligands

Epidermal growth factor (EGF), acting through its receptor EGFR, is a potent mitogen for epidermal keratinocytes. Not surprisingly, since cell proliferation is necessary for tumor promotion, EGFR signaling plays an important role in skin carcinogenesis. Many human cancers, including lung, breast, brain, and ovarian cancers, also have dysregulated EGFR expression or activity due to gene amplification, mutations or overexpression of ligands [100,101,102,103].

**Figure 2:**
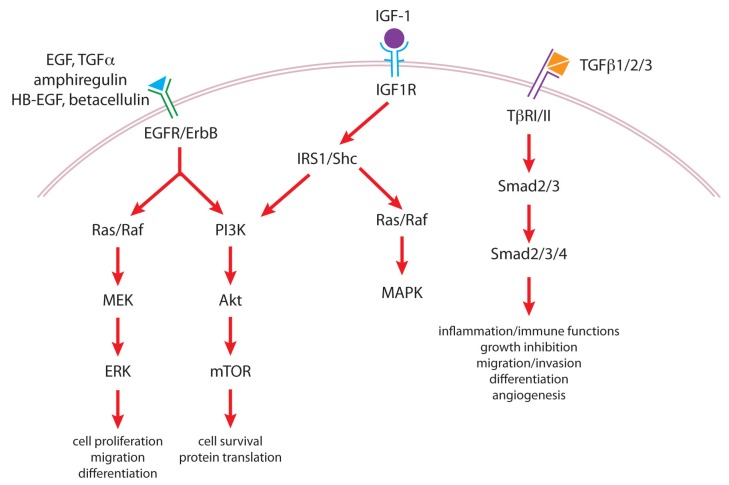
Growth factor signaling pathways important in tumor promotion. Epidermal growth factor (EGF) and other ligands of the EGF receptor (EGFR)/ErbB family of receptors, including transforming growth factor-α (TGFα), amphiregulin, heparin-binding EGF-like growth factor (HB-EGF) and betacellulin, activate signaling of both the Ras/Raf/mitogen-activated protein kinase (MAPK) and extracellular signal-regulated kinase (ERK) kinase (MEK)/ERK and phosphatidylinositol-3-kinase (PI3K)/Akt/mammalian target of rapamycin (mTOR) pathways. Insulin-like growth factor-1 (IGF-1) signals through its receptor IGF1R, which phosphorylates insulin receptor substrate-1 (IRS1) and SH2-containing protein (Shc) as well as other substrates leading to the activation of both Ras/Raf/MAPK and PI3K/Akt/mTOR pathways. Members of the transforming growth factor-β (TGFβ) family (TGFβ1, TGFβ2 and TGFβ3) bind to TGFβ receptor type II (TβRII) causing the recruitment of the type I receptor (TβRI), which phosphorylates Smad2 and Smad3 allowing them to form a complex with Smad4 that then translocates to the nucleus to modulate gene expression.

EGFR (or ErbB1) is a member of the ErbB/HER family of transmembrane receptor tyrosine kinases, which also includes, ErbB2 (HER2 or Neu), ErbB3 and ErbB4. Ligands bind to the extracellular domain of EGFR, ErbB3 or ErbB4, which induces homodimerization and/or heterodimerization with ErbB2 and activation of the kinase domain in the cytoplasmic tail [101]. Receptor dimerization results in autophosphorylation of key tyrosine residues within the cytoplasmic domain, which form docking sites for downstream adapter and signaling proteins, and subsequent tyrosine phosphorylation of the docked proteins [101,103]. Adaptor/signaling protein recruitment and phosphorylation leads to activation of multiple signaling pathways including MAPK, PI3K/Akt and PLCγ/PKC pathways (Figure 2). Cellular context, as well as the ligand bound and dimer composition leading to formation of specific docking sites on specific phosphorylated tyrosines determines which particular pathway(s) are activated [104]. Signal transduction from the ErbBs leads to transcriptional activation of specific gene programs that affect cell proliferation, migration, differentiation, adhesion, and apoptosis [104].

The ErbB ligands are all characterized by a consensus sequence of six conserved cysteines that form three intramolecular disulfide bonds known as the EGF motif, which is necessary for binding to ErbB receptors [105]. Ligands include EGF, transforming growth factor-α (TGFα), heparin-binding EGF-like growth factor (HB-EGF), amphiregulin, epiregulin, betacellulin and neuregulins (or heregulins). All ligands are synthesized as large transmembrane precursors that are released as mature growth factors by cell surface proteases [104,105]. The ligands show preferential binding to different ErbB receptors such that EGF, TGFα and amphiregulin are specific for EGFR; HB-EGF, betacellulin and epiregulin bind to either EGFR or ErbB4; while the neuregulins bind to ErbB3 and ErbB4 [101,104]. ErbB2 has no known ligand, but heterodimerizes with the other ErbBs leading to potent and prolonged activation of downstream signaling pathways [101,104].

#### 3.1.2. Role of EGFR/ErbB Signaling in Skin Tumorigenesis

The role of EGFR/ErbB signaling in mouse skin tumor promotion has been extensively studied. Single and multiple applications of various skin tumor promoters up-regulate epidermal expression of TGFα [106,107]. TGFα expression is elevated in mouse skin tumors induced by DMBA initiation and promoted with either TPA or chrysarobin [108]. Transgenic mice that overexpress TGFα in the skin *via* either a K14 or keratin 1 (K1) promoter develop papillomas upon wounding or with TPA treatment without carcinogen initiation [109,110,111], demonstrating that high expression levels of TGFα can act as an initiating event, as well as enhance tumor promotion. Conversely, wa-1 mice with a spontaneous inactivating mutation in the TGFα gene show only modest epidermal hyperplasia after multiple TPA treatments and are resistant to two-stage skin carcinogenesis [112]. However, TPA induces the mRNA expression of other EGFR ligands, amphiregulin and HB-EGF, in wa-1 mice [112]. TGFα null and wa-1 keratinocytes transformed with the v-H-*ras* oncogene and grafted onto nude mice give rise to skin tumors (mostly papillomas) similar to that of keratinocytes from BALB/c or C57BL mice (wild-type counterparts to wa-1 or TGFα null, respectively) [113]. The latter results suggest that TGFα is not necessary for v-H-ras induction of skin tumors and that other EGFR ligands may also contribute to tumorigenesis.

Early studies showed that various skin tumor promoters including TPA, chrysarobin, thapsigargin, palytoxin and UVB irradiation, as well as TCDD inhibit the ability of EGF to bind to its receptor in short-term experiments in cultured cells (reviewed in [114]). However, topical treatment *in vivo* with either TPA or chrysarobin significantly increases EGF binding to EGFR in the long term [106]. Multiple applications of TPA, chrysarobin or okadaic acid result in increased tyrosine phosphorylation (activation) of EGFR and elevated levels of activated EGFR are found in skin papillomas [115]. Similarly, UV exposure rapidly activates EGFR [116]. Tyrosine kinase inhibitors, which block EGFR signaling, are able to inhibit TPA-induced epidermal hyperplasia and cell proliferation [115]. UV-induced epidermal hyperplasia is completely abrogated in EGFR null mice, while UV-induced apoptosis is enhanced [116]. Grafts of EGFR-null keratinocytes transformed with v-H-*ras* yield skin tumors that are much smaller than wild-type keratinocytes, which demonstrates that EGFR signaling contributes to tumor growth [117]. EGFR-null keratinocytes immortalized with human papilloma virus (HPV)-16 are unable to form tumors when grafted onto nude mice, while HPV16-immortalized wild-type keratinocytes form tumors to some extent (17%), a few of which progress to carcinomas [118]. Taken together, these results demonstrate that EGFR signaling is important for keratinocyte proliferation, survival and skin tumor promotion.

In addition to EGFR, ErbB2 and ErbB3 are expressed in mouse keratinocytes in culture and in mouse epidermis *in vivo* [119]. TPA treatment results in increased tyrosine-phosphorylated ErbB2 and formation of EGFR:ErbB2 heterodimers in the epidermis [119]. Similarly, K14.TGFα transgenic mice show activation of ErbB2 in the skin [119]. K5.ErbB2 transgenic mice display both follicular and epidemal hyperplasia and develop spontaneous skin tumors, some of which progress to squamous cell carcinomas [120]. The epidermis of these mice has elevated levels of EGFR as well as ErbB2, shows EGFR:ErbB2 and ErbB2:ErbB3 heterodimer formation and tyrosine phosphorylation [120]. ErbB2 transgenic mice are more sensitive to TPA-induced epidermal proliferation and two-stage skin tumorigenesis compared to wild-type mice [120]. Thus, ErbB2 signaling *via* heterodimerization with EGFR and/or ErbB3 strongly enhances epidermal growth and skin tumorigenesis.

### 3.2. Insulin-like Growth Factor-1 (IGF-1) and Receptor

IGF-1 and IGF-2 are single-chain polypeptides that structurally resemble proinsulin and both have mitogenic activity [121,122]. Expression of IGF-1 is regulated primarily by pituitary-derived growth hormone and its expression and secretion by the liver is responsible for the majority of circulating levels [121,123]. Both IGF-1 and IGF-2 bind to and signal through the IGF-1 receptor (IGF-1R), which is a receptor tyrosine kinase consisting of an extracellular α subunit homodimer and a transmembrane β subunit homodimer [121,124]. IGF-1 binding to the extracellular α subunits activates the cytoplasmic tyrosine kinase domains in the β subunits resulting in autophosphorylation on tyrosine residues, which allows docking and phosphorylation of its major substrates, insulin receptor substrate-1 (IRS-1), IRS-2 and Shc [122,124]. IGF-1R signaling leads to the activation of the PI3K/Akt pathway through phospho-IRS-1 or the activation of the Ras-Raf-MAPK pathway either through phospho-IRS-1 or phospho-Shc (Figure 2) [124]. IGF-1/IGF-1R signaling results in stimulation of cell proliferation and/or cell survival/inhibition of apoptosis [122].

IGF-1/IGF-1R signaling has been implicated in the promotion of carcinogenesis in both humans and animals (reviewed in [121,123]). In the mouse skin model, IGF-1 mRNA levels are elevated consistently in papillomas and carcinomas, while IGF-1R mRNA is up-regulated in some but not all tumors, mostly carcinomas [125]. Unlike TGF-α, single or repeated applications of the tumor promoter TPA fails to alter IGF-1 expression [125]. Transgenic mice that overexpress IGF-1 in the epidermis *via* either a K1 or K5 promoter are hypersensitive to the proliferative effects of TPA and develop skin tumors without carcinogen initiation [126,127]. Using of the two-stage protocol with DMBA plus various tumor promoters, the K1.IGF-1 transgenic mice develop more tumors/mouse more rapidly than their non-transgenic littermates [128]. K5.IGF-1 transgenic mice also form skin tumors with only DMBA initiation [127]. Together these data demonstrate that IGF-1 overexpression contributes to both initiation and promotion of skin tumorigenesis. On the other hand, liver-specific IGF-1 deficient (LID) mice, which have ~75% reduction in circulating IGF-1 levels, have a reduced response to two-stage skin carcinogenesis and less activation of both IGF-1R and EGFR in response to TPA compared to wild-type mice [129]. Thus, circulating IGF-1 also contributes to skin carcinogenesis.

### 3.3. Transforming Growth Factor-β (TGFβ) and Receptors

#### 3.3.1. TGFβ Signaling

The TGFβ family includes three members, TGFβ1, TGFβ2 and TGFβ3. All three TGFβs are secreted as latent complexes, which consist of dimers of their C-terminal mature peptide and N-terminal pro domain in association with a latent TGFβ-binding protein that targets and sequesters TGFβ in the extracellular matrix [130]. TGFβ must be released from the matrix and activated by proteolysis or conformational change in order to bind to and signal through its receptors [130,131]. Active TGFβ initially binds to homodimeric TGFβ type II receptor (TβRII), which results in recruitment of a dimer of type I receptor (predominantly TβRI, also called ALK5) to form a heteromeric complex [132,133]. The TβRII cytoplasmic serine/threonine kinase domain phosphorylates the GS domain of TβRI, which results in activation its kinase domain and autophosphorylation, as well as phosphorylation of downstream targets, in particular Smad2 and Smad3 [131,132,133]. Phosphorylated Smad2/3 is then able to form a complex with Smad4, which then translocates to the nucleus, where the Smads directly and indirectly regulate gene transcription (Figure 2) [131,132,133].

#### 3.3.2. Multiple Roles of TGFβ Signaling in Tumor Promotion

The role of TGFβ signaling in cancer and tumor promotion is multifaceted and complex. TGFβ is growth inhibitory to epithelial cells and induces apoptosis in several cell types and so acts as a tumor suppressor [131]. Growth inhibition by TGFβ is due to repression of c-*myc* transcription *via* a Smad3/4, E2F4/5 and p107 complex forming on its promoter, and to up-regulation of the transcription of cyclin-dependent kinase inhibitors p15^ink4b^ and p21^cip1^ *via* interactions between Smads and Sp1 on their promoters [134]. Malignant progression is often associated with development of resistance to the growth inhibitory effects of TGFβ, and a number of different human cancers have inactivating mutations in genes for TGFβ receptors, Smad2 or Smad 4 [131,133,135]. On the other hand, many cancers overexpress TGFβ1 and TGFβ signaling can promote carcinogenesis and cancer progression by inducing angiogenesis, cancer cell invasion into the extracellular matrix, epithelial-to-mesenchymal transition (EMT) and metastasis, as well as by repressing immunosurveillance [131,133].

TGFβ also has contradictory roles in inflammation and immune function. TGFβ has anti-inflammatory actions since TGFβ1 null mice develop multifocal inflammatory disease resulting in early death [136,137]. However, TGFβ1 is also a potent chemoattractant of neutrophils [138] and transgenic mice that overexpress TGFβ1 in the skin show inflammatory cell infiltration associated with up-regulated expression of proinflamatory cytokines and chemokines, such as interleukin-1 (IL-1), TNFα, interferon-γ (IFNγ), monocyte-chemotactic protein-2 (MCP-2) and others [139]. Tumor-induced TGFβ suppresses immune surveillance by inducing IL-17 expression in CD8+ T cells [140]. Thus, although TGFβ signaling can be anti-inflammatory and therefore tumor suppressive, its role as a chemoattractant and in immune suppression can contribute to promotion of tumorigenesis.

#### 3.3.3. Role of TGFβ Signaling in Skin Tumorigenesis

In the mouse skin model, TGFβ1 mRNA is transiently induced in response to several different tumor promoters and is constitutively overexpressed in squamous cell carcinomas [135,141,142]. However, loss of TGFβ1 immunostaining in papillomas has been correlated with an increased risk of malignant conversion [143,144]. Two-stage carcinogenesis experiments with transgenic mice that overexpress TGFβ1 in the epidermis *via* either K6 or K10 promoters results in formation of fewer papillomas, but increased conversion to carcinomas and progression to aggressive spindle cell carcinomas with elevated TGFβ3 expression [145]. In an inducible transgenic model, induction of TGFβ1 expression after papilloma development promotes progression to carcinomas that metastasize to lymph nodes [146]. Transgenic overexpression of TGFβ1 in papillomas is associated with up-regulated matrix metalloproteinase (MMP) expression, loss of membrane-associated E-cadherin/catenin complex and increased angiogenesis [146].

Disruption of TGFβ signaling by overexpression of a dominant negative TβRII in the epidermis *via* either K5 or loricrin promoters results in more skin tumor development in two-stage carcinogenesis protocols, with accelerated and greatly increased incidence of carcinoma development [147,148]. Both premalignant and malignant tumors from these mice have increased angiogenesis along with elevated expression of vascular endothelial growth factor (VEGF) and reduced expression of thrombospondin-1 [148]. While TGFβ1 transgenic mice develop spindle cell carcinomas demonstrating EMT, tumors from bigenic mice that overexpress both TGFβ1 and dominant-negative TβRII do not, which demonstrates that TGFβ signaling in the epithelial cells is required for EMT, while induction of invasion and metastasis is not [149]. Together these results suggest that TGFβ signaling is tumor suppressive at early stages *via* its growth inhibition of carcinogen-initiated epithelial cells; but as tumor cells become resistant to TGFβ’s growth inhibitory effects, TGFβ produced by the tumor or other cells acts on the stroma to enhance malignant progression *via* induction of VEGF, MMPs and down-regulation of thrombospondin-1.

Smad null mice further demonstrate the opposing actions of TGFβ signaling on tumor promotion in mouse skin. Smad3 null mice are resistant to TPA induction of epidermal proliferation and two-stage skin carcinogenesis with reduced infiltration of tumor-associated macrophages and expression of IL-1β and MCP-1 in papillomas compared to wild-type mice [150]. In contrast, mice with skin-specific knockout of Smad4 have epidermal hyperplasia and develop spontaneous malignant squamous cell carcinomas, which is associated with inactivation of phosphatase and tensin homolog deleted on chromosome 10 (PTEN), activation of Akt and nuclear accumulation of cyclin D1 [151]. Together these results suggest that Smad3 signaling is necessary for TGFβ’s proinflammatory effects, while Smad4 is important for TGFβ’s growth inhibitory and pro-apoptotic effects.

## 4. Proinflammatory Cytokines and Prostaglandins

### 4.1. Tumor Necrosis Factor-α (TNFα)

#### 4.1.1. Receptors and Signaling

**Figure 3:**
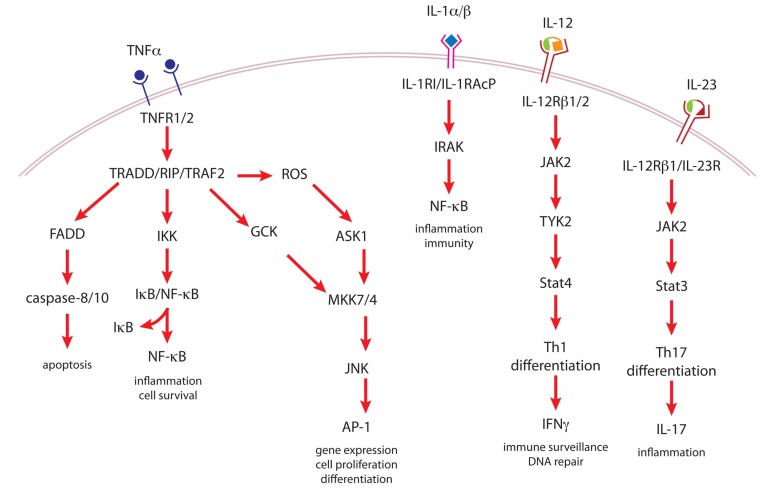
Cytokine signaling pathways important in tumor promotion.

TNFα is one of the critical cytokines mediating tumor promoter-induced inflammation. TNFα is synthesized as a type II transmembrane pro-peptide that forms a homotrimer and is released as a soluble factor upon cleavage by the metalloprotease TNFα-converting enzyme (TACE) [152,153]. TNFα signals through two homotrimeric transmembrane receptors, TNFR1 and TNFR2. TNFR1 is expressed constitutively in most tissues and preferentially binds soluble TNFα, while TNFR2 expression is restricted primarily to hematopoietic and immune cells and is activated only by membrane-bound TNFα [152,154]. Ligand-bound TNFRs recruit multiple adaptor proteins that activate multiple signaling pathways. One adaptor protein is TNFR1-associated death domain protein (TRADD), which interacts with the cytoplasmic death domain of TNFR1 and serves to recruit binding of receptor-interacting protein kinase (RIP) and TNFR-associated factor-2 (TRAF2) to the receptor complex [154]. On the other hand, TRAF2 homodimers or TRAF1/2 heterodimers bind directly to TNFR2 to activate downstream signaling [154,155]. TNFα activation of TNFR1 initiates activation of NF-κB signaling by TRAF2 bound to TNFR1 *via* TRADD recruiting the IκB kinase (IKK) complex [154]. Then RIP activation of the IKKs releases NF-κB from its inhibitory complex with IκB [154]. TNFα stimulation of NF-κB transcriptional activity also occurs through PKCζ and PI3K/Akt phosphorylation of the transactivation domain of the NF-κB p65/c-Rel subunit [154,156], as well as Akt phosphorylation of IKKα (Figure 3) [157]. NF-κB signaling leads to induction of a variety of antiapoptotic factors.

Another TNFR1-mediated signaling pathway is activation of the JNK cascade. TNFR1/TRADD/TRAF2 complex interacts with germinal center kinase (GCK) family members, which results in activation of the MAPK kinase kinases MKK7 and MKK4 upstream of JNK [154]. In addition, TNFR1/TRAF2-dependent generation of ROS leads to activation of another MAPK kinase kinase, apoptosis signal-regulating kinase-1 (ASK1) that then associates with TRAF2 and phosphorylates MKK7 [154]. Finally, activated JNK phosphorylates the AP-1 transcription factor component, c-Jun, leading to transcriptional up-regulation of genes involved in proliferation, differentiation and apoptosis (both pro- and anti-apoptotic proteins).

Tumor necrosis factor-α (TNFα) binds to either of its receptors (TNFR1 and TNFR2), which recruit multiple adaptor proteins including TNFR1-associated death domain protein (TRADD), receptor-interacting protein kinase (RIP) and TNFR-associated factor-2 (TRAF2). Activation of TNFR1/TRADD signaling induces apoptosis through Fas-associated death domain protein (FADD) and caspase-8 and -10. On the other hand, TNFR1/TRADD/TRAF2/RIP causes activation of inhibitor of κB (IκB) kinase (IKK), which in turn phosphorylates and releases IκB from nuclear factor-κB (NF-κB) to activate NF-κB signaling. In addition TNFR1/TRADD/TRAF2 activates the MAPK kinase kinases 7 and 4 (MKK7 and MKK4)/c-Jun N-terminal kinase (JNK)/activator protein-1 (AP-1) signaling cascade either by interacting with germinal center kinase (GCK) or *via* generation of reactive oxygen species (ROS) and activation of apoptosis signal-regulating kinase-1 (ASK1). Interleukin-1α and β (IL-1α and IL-1β) bind to their receptor IL-1RI, which recruits IL-1R accessory protein (IL-1RAcP) and activates IL-1R-associated kinase (IRAK) that then activates the NF-κB signaling pathway. IL-12 and IL-23 are heterodimers with a common p40 subunit (green) disulfide-linked with either a p35 subunit (IL-12) or a p19 subunit (IL-23). IL-12 binds to its receptor consisting of IL-12Rβ1 and IL-12Rβ2, which associates with Janus kinase (JAK) family members JAK2 and TYK2 that then phosphorylate and activate signal tranducer and activator of transcription 4 (Stat4) leading to Th1 differentiation of T-helper cells. IL-23 binds to IL-12Rβ1 coupled with IL-23R, which leads to JAK2 activation of Stat3 and Th17 differentiation of T-helper cells.

Like other members of the death domain-containing receptors, activation of TNFR1 is able induce apoptosis through association with Fas-associated death domain (FADD) adaptor protein (*via* TRADD), which recruits and activates caspases-8 and -10 [154]. However, TNFα-induced apoptosis plays only a minor role in the overall response of cells to TNFα. TNFα’s proapoptotic signal is counteracted by up-regulation of NF-κB-dependent prosurvival genes, as well as by TNFR1/TRAF2 recruitment of cellular inhibitor of apoptosis proteins-1 and -2 (cIAP1/2), which are able to block activation of caspase-8 [154]. However, in the absence of NF-κB activation, the primary effect of TNFα signaling is induction of apoptosis [158].

TNFα signaling is also modified by the cleavage and shedding of the extracellular domain of TNFRs, which can act as soluble TNFα binding proteins that compete with binding to cell surface receptors, but that also stabilize trimeric TNFα and serve as a slow-release reservoir [152,159]. Thus, there are many levels of regulation of TNFα signaling and crosstalk with other signaling pathways. The net effect of TNFα depends on cell context, presence of specific intracellular adaptor proteins and interaction with other signaling pathways to induce proinflammatory mediators, prosurvival proteins, oxidative stress and/or cell death by apoptosis or necrosis.

#### 4.1.2. Role of TNFα in Tumor Promotion

In the mouse skin carcinogenesis model, TPA induction of TNFα expression in the epidermis is responsible for the early phases of TPA-induced inflammation and skin edema [160,161]. TNFα null mice are resistant to skin carcinogenesis induced by DMBA initiation with TPA or okadaic acid promotion, or by repeated DMBA exposure [161,162], which clearly demonstrates the critical role of TNFα in skin tumor promotion. However, TNFα has little effect on the later stages of carcinogenesis since there are similar rates of malignant conversion in TNFα null and wild-type mice [161]. Both TNFR1 and TNFR2 are expressed on epidermal keratinocytes and both play roles in skin tumor promotion [163]. TNFR1 null mice are more resistant to DMBA/TPA-induced tumor development than TNFR2 null mice, which indicates that TNFR1 is the major mediator of TNFα-promoted tumorigenesis [163]. Likewise, TNFR1 and TNFR2 null mice on a SKH-1 hairless background are resistant to UV-induced skin tumorigenesis, with TNFR1 null mice showing a complete lack of inflammatory cell infiltration after UV irradiation [164]. TPA induction of PKCα membrane translocation, down-regulation and AP-1 activation are substantially delayed in TNFα null mice, which correlates with reduced TPA induction of AP-1-regulated genes: granulocute/macrophage-colony stimulating factor (GM-CSF), MMP-3 and MMP-9 [165]. In addition, keratinocytes from TNFα null mice have reduced levels of integrin αvβ6, which is important for keratinocyte migration and induction of MMP-9 [166]. TPA-induced expression of c-Jun is transient and its phosphorylation is reduced in the epidermis of TNFR1 and TNFR2 null mice compared to wild-type mice [163]. Induction of MMP-3 by TPA is also reduced in both TNFR1 and TNFR2 null mice, while induction of GM-CSF is reduced more in TNFR2 null mice, but MMP-9 more in TNFR1 null mice compared to wild-type mice [163]. Thus, TNFα signaling through its receptors, TNFR1 and TNFR2, induces transcription of different AP-1-regulated genes that contribute to inflammation, keratinocyte migration and carcinogenesis, which makes TNFα a critical mediator of skin tumor promotion.

### 4.2. Interleukins

Interleukins were initially identified (and named) as a family of structurally related proteins that are secreted by leukocytes and act on other leukocytes [167]. Interleukins are now recognized as members of the large cytokine family of proteins that are produced by and act on many cell types besides just leukocytes. Although the interleukin/cytokine family is large and its members have pleiotropic effects in inflammation and immune responses, only several interleukins with clear roles in skin tumor promotion are reviewed here.

#### 4.2.1. Interleukin-1 (IL-1)

The IL-1 family of ligands consists of IL-1α (new name, IL-1F1), IL-1β (IL-1F2) and IL-1 receptor antagonist (IL-1Ra or IL-1F3). IL-1α and IL-1β, both of which lack signal peptides, are agonists and are synthesized as precursors that are processed to their mature forms by calpain and IL-1β-converting enzyme (ICE or caspase-1), respectively [168,169]. While IL-1β is only active as a secreted mature protein, IL-1α is primarily active in cell-associated forms, either in an intracellular precursor form or as a membrane-bound processed form [168]. In contrast, IL-1Ra, which has a signal peptide and is secreted *via* the endoplasmic reticulum-Golgi pathway (sIL-1Ra), is a physiological antagonist of IL-1 signaling [168]. An intracellular form of IL-1Ra (icIL-1Ra), which lacks a signal peptide, is constitutively expressed in epidermal keratinocytes and intestinal epithelial cells and blocks intracellular IL-1α signaling [169].

There are two transmembrane IL-1 receptors (IL-1Rs): type I (IL-1RI), which is the signaling receptor for both IL-1α and IL-1β, and type II (IL-1RII), which does not transduce a signal [169]. IL-1RII, which has higher affinity for IL-1β than IL-1α or IL-1Ra, has been called a decoy receptor and acts as a sink for IL-1β [169,170]. Upon IL-1 binding to IL-1RI, a transmembrane receptor accessory protein (IL-1RAcP), which is an IL-1RI homolog, is recruited to the complex, followed by activation of IL-1R-associated kinase (IRAK) and propagation of signal, often through activation of NF-κB (Figure 3) [168,170]. IL-1Ra bound to IL-1RI as well as ligand-bound IL-1RII, which has a very short cytoplasmic tail, are unable to recruit IL-1RAcP and IRAK to the complex and thus, no signal is produced [168]. In addition, soluble forms of IL-1RI and RII are produced and act as buffers of IL-1 signaling. Thus, under homeostatic conditions, IL-1 signaling is kept in check by the presence of IL-1Ra, membrane-bound IL-1RII and soluble IL-1Rs [168].

IL-1 is one of a trio of cytokines, along with TNFα and IL-6, that is secreted by monocytes and macrophages, which drive the acute phase of inflammation [168,171]. Many cell types produce IL-1 after stimulation by microorganisms, cytokines or other environmental insults. Then IL-1α activates adjacent cells (or IL-1β on distant cells) to induce the expression of additional pro-inflammatory genes, including IL-6, COX-2 and inducible nitric oxide synthase (iNOS) [168,172].

In the skin, IL-1 plays multiple roles. Cultured keratinocytes express IL-1α and IL-1β constitutively, which act autocrinely to stimulate growth [173]. IL-1α and IL-1β mRNA expression in keratinocytes is significantly induced further by exposure to TPA or UV [174]. Similarly, various skin tumor promoters including TPA, anthralin and mezerein induce IL-1α mRNA and protein expression in the epidermis *in vivo* [175,176]. However, tumor promoters such as TPA, anthralin and thapsigargin also up-regulate expression of the antagonist IL-1Ra in mouse skin keratinocytes *in vivo* and *in vitro* [177]. Blocking the activity of IL-1α with intradermal injections of a neutralizing antibody or by transgenic overexpression of IL-1RII (*via* K14 promoter) inhibits TPA-induced vascular permeability, inflammatory cell infiltration and epidermal hyperplasia, which demonstrates the central role of IL-1α in mediating these tumor promoter-related events [178,179]. Conversely, transgenic mice that overexpress IL-1α in basal keratinocytes (K14 promoter) develop spontaneous inflammatory skin lesions, as well as dermal neutrophil infiltration even in nonlesional skin [180], which demonstrates the potent proinflammatory role of IL-1. Psoriatic or TPA-treated skin have highly up-regulated expression of the non-signaling IL-1RII in the proliferating basal layer [181], while IL-1RI and IL-1Ra are highly expressed in the differentiated cells [182,183], which suggests a complex regulation of IL-1 signaling in the epidermis to promote both proliferation and differentiation. IL-1β treatment has been shown to activate MAPKs p38 and ERK, leading to phosphorylation of cAMP-response element binding protein (CREB) and transcriptional up-regulation of the AP-1 factor c-Fos [184]. Blocking this pathway inhibits IL-1β-induced proliferation [184].

In the skin carinogenesis model, a single carcinogenic dose of DMBA induces IL-1α mRNA and protein in the skin and five doses of DMBA increases serum IL-1α levels similar to that induced by TPA [185]. IL-1α and IL-1Ra proteins are expressed primarily in the non-proliferating suprabasal layers of the epidermis and papillomas [175,177], with IL-1Ra expression up-regulated in papillomas and carcinomas compared to normal skin [177]. Stable overexpression of icIL-1Ra in a mouse skin carcinoma cell line results in down-regulated COX-2 expression and slower *in vitro* and *in vivo* growth [186]. These results indicate that IL-1 is contributing to malignant cell proliferation, which icIL-1Ra is able to inhibit. DMBA/TPA induction of papillomas is increased in IL-1Ra null compared to wild-type or IL-1α null mice, which suggests that unopposed IL-1 signaling promotes inflammation-mediated skin tumorigenesis [172]. However, K14.IL-1α transgenic mice are completely resistant to skin tumorigenesis induced by DMBA/TPA or by overexpression of activated H-ras (Tg.AC transgenic mice), perhaps due to IL-1α activation of the innate immune system to eliminate initiated cells [187]. On the other hand, K14.IL-1α transgenic mice more rapidly develop carcinomas *de novo* without going through a papilloma stage using a complete carcinogenesis protocol [187]. Thus, as seen with other factors, IL-1 has multiple and at times, contradictory roles in tumor promotion.

#### 4.2.2. Interleukin-12 (IL-12) and Interleukin-23 (IL-23)

IL-12 is a disulfide-linked heterodimer consisting of a p40 subunit that is homologous to several cytokine receptors, such as the IL-6 receptor IL-6R, and a p35 subunit that is homologous to other cytokines, such as IL-6 [188]. IL-23 is also a heterodimer using the same p40 receptor-like subunit linked to a p19 subunit, which is closely related to IL-12p35 [189]. Both IL-12 and IL-23 are expressed primarily by activated dendritic cells, macrophages and monocytes [189]. The receptor for IL-12 consists of two transmembrane subunits, IL-12Rβ1 and IL-12Rβ2, which are physically associated with JAK2 and another Janus kinase member TYK2, respectively [167]. On the other hand, IL-23 uses IL-12Rβ1 coupled with a novel IL-23R subunit, which also signals through JAK/Stat [189]. While IL-12 induces a strong activation of Stat4 and a weaker activation of Stat3, the reverse is true of IL-23 (Figure 3) [189]. The IL-12Rβ receptors are expressed primarily on natural killer cells and on T cells, and their activation by IL-12 induces the differentiation of naïve T helper cells to the Th1 phenotype and expression of IFNγ [167,188,189]. On the other hand, IL-23R is expressed at low levels on monocytes, macrophages and dendritic cells as well as predominantly on natural killer cells and T cells, and IL-23 induces T helper cells to differentiate along the Th17 lineage and express IL-17 [190,191].

IL-23 mRNA, as well as IL-17, is overexpressed in a number of different human cancers compared to adjacent normal tissues [191]. IL-23p19 protein is localized within the tumor tissue as a result of infiltrating dendritic cells and macrophages [191]. IL-23p19 null mice, as well as p40 null mice, are resistant to DMBA/TPA induction of skin tumorigenesis [191], which supports a role for IL-23 in tumor promotion. On the other hand, IL-12p35 null mice develop papillomas earlier and more frequently than wild-type mice [191]. IL-17 is highly expressed in the hyperplastic skin of wild-type and p35 null mice, but is barely detectable in p19 and p40 null mice, which correlates with fewer infiltrating granulocytes and macrophages in the p19 and p40 null mice [191]. In contrast, cytotoxic CD8+ T cells are found in greater number in the epidermis and dermis of p19 and p40 null mice compared to p35 null and wild-type mice [191]. Thus, IL-12 acts as a tumor suppressor by inducing immune surveillance and anti-tumor responses, while IL-23 promotes skin tumorigenesis by driving inflammation and reducing immune surveillance.

In addition, IL-12 plays a key role in preventing UV-induced skin carcinogenesis. Application of IL-12 prevents UV-induced immune suppression, and this is dependent on IL-12 induction of the removal or repair of UV-damaged DNA [192]. IL-12p35 and IL-12p40 null mice are more sensitive to UV-induced skin carcinogenesis, with reduced repair of UV-induced DNA damage, more tumors per mouse, tumors that grow more rapidly and greater malignant conversion of papillomas to carcinomas than wild-type mice [193,194]. UV-induced tumors from p35 null mice have increased angiogenesis and up-regulated expression of pro-inflammatory IL-6 and IL-23 compared to tumors from wild-type mice [195]. Thus, IL-12 counteracts UV-induced immunosuppression, inflammation and skin carcinogenesis [192].

### 4.3. Prostaglandins

#### 4.3.1. Cyclooxygenases (COXs)

The COX enzymes catalyze the initial and rate-limiting step of arachidonic acid metabolism to bioactive prostaglandins (PGs), prostacyclins and thromboxanes. COX-1 and COX-2 are the two major isoforms and are encoded by separate genes. COX-1 is constitutively expressed in most tissues and its expression usually does not vary much in the adult animal [196]. The PG products of COX-1 are involved in normal physiological functions, such as maintenance of the gastric mucosa and regulation of renal blood flow [197]. On the other hand, COX-2 expression is undetectable in most unperturbed adult epithelial tissues except kidney and brain, but is highly inducible by various mitogenic and inflammatory stimuli, including growth factors, cytokines, hormones, serum, hypoxia, bacterial endotoxins, tumor promoters and UV light [196,198,199]. PGs produced by COX-2 are involved in pathophysiological functions such as inflammation, fever, pain, wound repair, angiogenesis, vasodilation and vascular permeability [196,200].

Up-regulated expression of COX-2 is found in multiple human cancers including colon, breast, prostate and skin [201,202,203]. In the mouse skin model, COX-2 is constitutively overexpressed in papillomas and carcinomas, which is accompanied with high levels of PGE_2_ and PGF_2__α_ [204]. COX-2 expression and PGE_2_ production are transiently induced in the epidermis *in vivo* and keratinocytes in culture by skin tumor promoters such as TPA, anthralin, okadaic acid and UV [198,204,205,206,207,208,209]. The release of the COX-2 substrate, arachidonic acid, from the membranes is also induced by TPA *via* PKC activation of phospholipase A_2_ (PLA_2_) [210]. COX-2 is transcriptionally up-regulated *via* multiple pathways. As expected, TPA induction of COX-2 expression requires PKC activation [206,211]. p38 MAPK activation of the transcription factors Sp1/Sp3 mediates EGF/EGFR-induced COX-2 expression [212]. In addition, nuclear ErbB2 binds to and activates the COX-2 promoter directly [213]. Induction by TNFα utilizes NF-κB and NF-IL6 binding sites in the COX-2 promoter [214]. Upstream stimulatory factors (USFs) and CCAAT/enhancer-binding protein (C/EBP) binding to COX-2 promoter E-box and NF-IL6 sites, respectively, are important for the constitutive up-regulation of COX-2 in mouse skin carcinoma cells [215]. UV induction of COX-2 expression also involves several pathways. UV-induced ROS leads to EGFR activation, which in turn activates Ras/Rac1/p38 and PI3K/Akt pathways ultimately resulting induction of COX-2 gene transcription *via* CREB binding to the CRE site in the COX-2 promoter [208]. UVB also converts intracellular tryptophan to its photoproduct FICZ, which binds to the AhR receptor, releasing it from Hsp90 and c-Src. Translocation of c-Src to the cell membrane activates the EGFR/Ras/Raf/ERK pathway to induce COX-2 transcription [90]. UVB-generated DNA damage up-regulates p53 leading to the induction of HB-EGF, which then activates EGFR/Ras/Raf/ERK to turn on COX-2 gene expression [216]. Finally, UVA wavelengths enhance the release of arachidonic acid from membrane lipid through the induction of ROS production, lipid peroxidation and activation of PLA_2_ [217]. In addition, UVA-generated singlet oxygen activates p38, which leads to the stabilization of COX-2 mRNA and thus, up-regulation COX-2 protein levels [217]. Elevated COX-2 protein levels and activation of PLA_2_ by UV and tumor promoters increases the production of PGE_2_, which in turn further enhances COX-2 expression in a positive feedback loop *via* activation of adenylate cyclase, increased cAMP levels, activation of PKA and CREB transcriptional activity [218].

Pharmacological and genetic approaches have demonstrated the critical importance of COX-2 expression and PGE_2_ production to tumor promotion. Topical application of various non-steroid anti-inflammatory drugs (NSAIDs), which are COX inhibitors, inhibit TPA induction of ornithine decarboxylase (ODC), epidermal proliferation and skin tumorigenesis [205,219,220]. Similarly, topical or dietary administration of NSAIDs, in particular COX-2-selective inhibitors, inhibits UV induction of inflammation, epidermal proliferation, oxidative DNA damage and skin tumorigenesis [209,221,222,223,224]. In humans, regular use of NSAIDs has been shown to be associated with a reduced risk of developing actinic keratoses and skin squamous cell carcinomas [225], as well as colorectal cancer [226] and breast cancer [227]. However, because COX-2-selective inhibitors also have COX-2-independent effects [228], genetic manipulation of COX-2 expression has been used to definitively demonstrate that COX-2 and PGs contribute to tumor promotion.

Two-stage skin carcinogenesis is reduced by ~75% in COX-1 and COX-2 null mice compared to wild-type mice and is associated with premature keratinocyte differentiation [229]. This suggests that PGs produced by both isoforms of COX are necessary for TPA tumor promotion. On the other hand, UV-induced skin carcinogenesis is the same in COX-1 null as in wild-type mice [230], while deletion of even just one COX-2 allele significantly reduces UV-induced tumor development [231]. Acute UV irradiation of COX-2 heterozygous or homozygous null mice induces greater apoptosis and less proliferation in the epidermis than wild-type mice [231,232]. Thus, for UV-mediated tumorigenesis, COX-2, but not COX-1, expression is critically important.

Transgenic mice that overexpress COX-2 in the basal layer of the skin *via* a K5 or K14 promoter have elevated levels of epidermal PGs and are more sensitive to skin carcinogenesis induced by a single dose of DMBA than wild-type mice [233,234,235]. Likewise, K14.COX-2 transgenic mice on a SKH-1 hairless background show accelerated and greater UV-induced skin tumor development [231]. Stable overexpression of COX-2 in a human basal cell carcinoma cell line results in elevated PGE_2_ production, secretion of angiogenic factors and resistance to UV-induced apoptosis [236]. Taken altogether, these results suggest that COX-2-derived PGs act as endogenous skin tumor promoters *via* antiapoptotic, proliferative, inflammatory and angiogenic mechanisms.

#### 4.3.2. Prostaglandin E_2_ (PGE_2_) Receptors and Signaling

**Figure 4:**
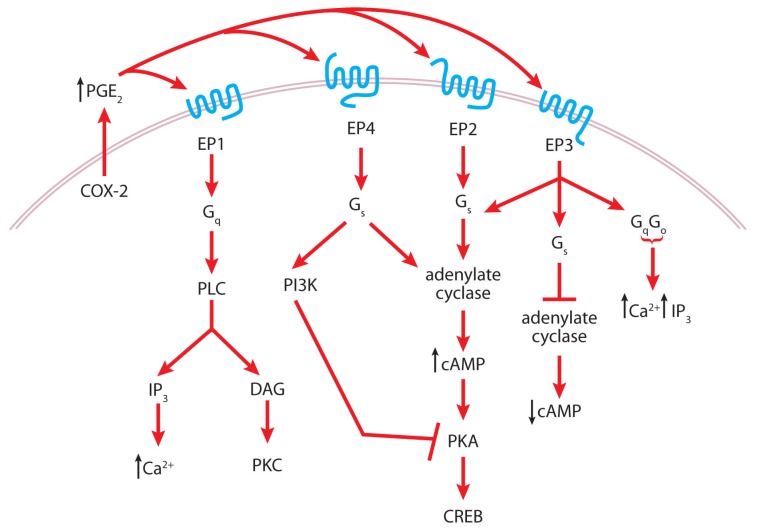
Cyclooxygenase-2 (COX-2) and prostaglandin E_2_ (PGE_2_) signaling in tumor promotion.

To understand the mechanisms by which COX-2 expression promotes tumorigenesis, attention has now turned to signaling by COX-2 products. The predominant COX-2-derived PG produced by the skin is PGE_2_. PGE_2_ induces keratinocyte proliferation *via* multiple signaling pathways including activation of EGFR, ERK, PI3K/Akt and PKA [237]. PGE_2_ binds to and activates four G protein-coupled receptors called EP1-EP4 [238]. Each EP receptor is coupled to different G proteins leading to activation of various downstream mediators (Figure 4). EP1 activation results in activation of PLC, leading to the generation of inositol phosphates and DAG, and elevation of intracellular calcium levels; EP2 and EP4 are both coupled to adenylate cyclase, which results in increased cAMP levels; and EP3, which has several splice variants, can activate or inhibit adenylate cyclase or increase intracellular calcium levels depending on the splice variant expressed [238,239,240]. Although both EP2 and EP4 signal through cAMP, which in turn activates PKA that phosphorylates CREB to enhance its transcriptional activity, EP4 can also signal through PI3K, which inhibits PKA activity [241].

In mouse skin, EP1 receptor expression has been shown by immunohistochemical staining to be low, while EP2 is patchy, EP3 is moderate and EP4 is undetectable [242]. In human skin, EP1, EP2, and EP3 expression is seen throughout the epidermis [243,244]. TPA treatment of mouse epidermis induces EP1 and EP2 expression and both are up-regulated in DMBA/TPA-induced papillomas and carcinomas ( [245] and S. M. Fischer, unpublished data). UV irradiation of SKH-1 mice strongly induces expression of EP1 primarily in the suprabasal layers, while EP3 expression becomes undetectable [242,246]. Similarly, in UV-induced skin tumors, EP1 mRNA and protein expression is elevated compared to normal skin, while EP3 expression is reduced [242]. All 4 EP receptors have been shown to be expressed in human skin squamous cell carcinomas by immunostaining, with up-regulated expression of EP1, EP2, and EP4 mRNAs compared to normal skin [242]. On the other hand, both mouse and human basal cell carcinomas show little or no detectable immunostaining of any of the EP receptors [242].

Induction of COX-2 expression during tumor promotion leads to increased production of PGE_2_. PGE_2_ signals through 4 seven-transmembrane G protein-coupled receptors EP1-4. EP1 coupled with G_q_ activates phospholipase C (PLC) leading to generation of inositol triphosphate (IP_3_) and diacylglycerol (DAG), which in turn increase intracellular calcium levels and activate protein kinase C (PKC), respectively. Both EP2 and EP4 are coupled with G_s_ and activate adenylate cyclase to increase cyclic AMP (cAMP) levels, which activate protein kinase A to phosphorylate cAMP response element binding protein (CREB), increasing its transcriptional activity. EP4 can also activate phosphatidylinositol-3-kinase (PI3K), which inhibits PKA. EP3 through its splice variants is coupled with G_s_, G_q_ or G_o_ leading to activation or inhibition of adenylate cyclase or increases in intracellular calcium and IP_3_ levels.

Topical treatment of mice with an EP1-selective antagonist partially inhibits UV-induced skin inflammation, increase in epidermal thickness and tumorigenesis, similar to that of a COX-2-selective inhibitor [246]. In addition, activation of the EP1 receptor with a selective agonist rescues malignant mouse keratinocytes from growth inhibition induced by the NSAID indomethacin [247]. Thus, EP1-mediated signaling contributes at least partially to UV-induced skin tumorigenesis.

EP2 and/or EP4 coupled to cAMP signaling have been shown by use of EP receptor-selective agonists to mediate PGE_2_ induction of proliferation in human keratinocyte cultures [248]. In two-stage skin carcinogenesis, EP2 null mice have lower tumor incidence and fewer tumors/mouse along with reduced TPA-induced epidermal hyperplasia, inflammatory cell infiltration, IL-1α expression and lower cAMP levels than wild-type mice [249]. Similarly, UV-induced blood flow, ear swelling and inflammatory cell infiltration are reduced in EP2 and EP4 null mice and in wild-type mice treated with an EP4 antagonist [250]. In addition, EP2 null mice have less epidermal proliferation and hyperplasia after UV irradiation [251] and reduced COX-2 induction and PGE_2_ production after TPA treatment [252]. On the other hand, overexpression of EP2 in the skin *via* a K5 promoter enhances sensitivity to DMBA/TPA-induced skin tumor development with increased tumor angiogenesis and TPA-induced hyperplasia and inflammation [245]. K5.EP2 transgenic keratinocytes have a greater induction of COX-2 expression after TPA or PGE_2_ treatment than wild-type keratinocytes and this correlates with elevated cAMP levels and CREB phosphorylation [252]. PGE_2_-induced proliferation is enhanced in EP2 transgenic compared to wild-type keratinocytes, while PGE_2_ is unable to induce proliferation in EP2 null keratinocytes [252]. EP2 signaling also enhances angiogenesis by directly affecting endothelial cell migration and survival. EP2 null endothelial cells show reduced cell motility in response to PGE_2_ and are more susceptible to apoptosis induced by serum starvation [253]. Both EP2 and EP4 agonists reduce the excessive apoptosis induced by UV irradiation in COX-2 null mice [254]. Thus, PGE_2_ signaling through the EP2 and/or EP4 receptors is tumor promoting *via* its effects on proliferation, cell survival and angiogenesis.

With regard to EP3, one group has shown that deletion of EP3 increases the latency time to first tumor and reduces the incidence of carcinomas [255], while another group found no differences in tumor development between EP3 null and wild-type mice [249]. Treatment of cultured human keratinocytes with an EP3 agonist does not rescue growth inhibition induced by indomethacin and instead inhibits proliferation [244,248]. These results along with the moderate expression of EP3 in the skin and its down-regulation after UV exposure and in UV-induced tumors suggest that EP3 probably is not playing a major role in skin tumor promotion.

Taken together, COX-2-generated PGE_2_ signaling through the EP2 receptor is probably the primary contributor to skin tumorigenesis by mediating effects on keratinocyte proliferation, survival, inflammation and angiogenesis. However, it is likely that EP1, EP2 and EP4 all contribute to skin tumor development, with each receptor transmitting a portion of the tumor-promoting signals of PGE_2_.

## 5. Other Molecular Mechanisms

### 5.1. Oxidative Stress Mechanisms

In the initial inflammatory response to injury or irritants such as tumor promoters, mast cells and leukocytes are recruited to the damaged site and generate a respiratory burst, *i.e.*, an increased oxygen uptake and release of free radicals and ROS [256]. The release of large amounts of superoxide anion from neutrophils and macrophages, primarily through the action of NADPH oxidases, is part of the cytotoxic defense against microorganisms and certain chemicals [257]. However, repeated exposures to tumor promoters create a chronic inflammatory state with a sustained release of ROS, which results in chronic oxidative stress. Free radicals and nonradical ROS such as hydrogen peroxide released by phagocytic cells can cause damage, such as DNA strand breaks, mutations, sister chromatid exchanges, protein modifications and lipid peroxidation, to adjacent epithelial cells [6,256]. In addition, keratinocytes themselves can also generate their own ROS through metabolism of xenobiotics such as PAHs, induction of cytochrome P450 enzymes by activation of AhR and activation of PPARs [258,259]. Even COX metabolism of arachidonic acid generates free radical intermediates [196]. ROS directly causes DNA damage, including the formation of 8-hydroxy-deoxyguanosine (8-OHdG) adducts, which can lead to G-to-T transversions and mutations [257]. In addition, protein modifications induced by free radicals/ROS can affect DNA repair capacity, transcriptional regulation, apoptosis, metabolism and cell signaling [256].

Epigenetic mechanisms of oxidative stress include effects on DNA methylation. The presence of 8-OHdG and *O^6^*-methylguanine adducts in CpG islands strongly inhibits methylation of the adjacent cytosine [8]. Additionally, ROS-generated single-strand DNA breaks can induce *de novo* methylation [8]. Changes in DNA methylation alter chromatin structure and DNA accessibility to the transcriptional machinery. Global hypomethylation during early stages and specific hypermethylation of normally unmethylated CpG regions are common epigenetic changes seen in human cancers [8].

Oxidative stress also affects intracellular signaling through proteins that are sensitive to redox homeostasis, which leads to activation of ERK1/2, Akt and NF-κB signaling pathways that promote cell survival as well as activation of JNK, p38 and p53 that can result in cell cycle arrest and apoptosis [257]. Oxidation of the active site of phosphatases, such as PTEN, inhibits their activity and inhibition of PTEN results in activation of Akt and its anti-apoptotic pathways [260]. UV-induced ROS leads to oxidative inhibition of receptor-type tyrosine phosphatase-κ, which normally keeps EGFR unphosphorylated and inactive [261]. Thus, UV exposure rapidly leads to ligand-independent activation of EGFR and downstream mitotic and anti-apoptotic signals [261].

Oxidative stress and generation of free radicals and ROS are important contributors to tumor promotion. The most direct evidence comes from the fact that free radical-generating compounds such as benzoyl peroxide and anthralin are complete skin tumor promoters [14,262]. Other skin tumor promoters, including phorbol esters, anthrones and mezerein, induce epidermal production of hydrogen peroxide and ROS [12,13]. Indirect evidence comes from studies demonstrating that various antioxidants are inhibitors of TPA tumor promotion, including butylated hydroxyanisole (BHA), green tea and grape seed polyphenols, silymarin, genistein, ascorbic acid and a superoxide dismutase mimetic [12,263,264,265,266]. The superoxide dismutase mimetic also suppresses TPA-induced keratinocyte proliferation without affecting apoptosis [266]. Porphyrin antioxidants inhibit TPA-induced neutrophil infiltration, hydrogen peroxide generation in the skin and epidermal ODC activity [267]. The ability of TPA to induce ROS in the skin of different strains of mice correlates with their sensitivity to TPA tumor promotion [12,13].

The PKC activator DAG is also induces ROS in the skin, suggesting PKC may be necessary for TPA induction of oxidative stress [13]. However, the anthrone class of skin tumor promoters does not activate PKC and their biological effects are thought to be mediated by their own oxidation and generation of various radical intermediates including superoxide. Anthrones such as anthralin and chrysarobin undergo oxidation and the rate of oxidation of different analogs correlates with their ability to induce ODC activity and to promote tumors [262].

Production of ROS and free radicals also play an important role in UV-induced skin carcinogenesis. In addition to signature UV-induced DNA mutations, G-to-T transversions, typical of 8-OHdG-related lesions, are often found in the *ras* oncogene and *p53* tumor suppressor gene in UV-induced mouse and human skin tumors [7]. p53 reduces the redox potential within a cell by inducing the expression of antioxidant genes, while lack of wild-type p53 increases oxidative stress in cells leading to oxidative DNA damage, increased mutation rate and karyotype instability [260,268]. Thus, UV-induced ROS contributes to tumor initiation, but UV-induced inflammation and generation of ROS also enhances skin tumor promotion as described above and to malignant progression by induction of genetic instability. Thus, the generation of free radicals and ROS is a major component of chemical- and UV-induced skin tumorigenesis.

### 5.2. Ornithine Decarboxylase (ODC)

ODC catalyzes the first step in polyamine biosynthesis converting ornithine to putrescine, which is further metabolized to spermidine and spermine [269]. ODC is a homodimer with two active sites that are formed from residues from both subunits [269]. Polyamine levels are tightly regulated and ODC expression is controlled transcriptionally and by protein degradation. The half-life of the ODC protein is ~17 min in epidermis [270] and degradation of ODC is mediated by binding of a protein, antizyme, to the ODC monomer, which inhibits enzymatic activity and directs it to the 26S proteasome without the necessity of ubiquitination [269]. There are four antizyme genes and all inhibit ODC activity [269]. High levels of polyamines up-regulate antizyme expression and also inhibit its degradation by ubiquitination [269]. Hormones, growth factors and tumor promoters, as well as the oncogene c-Myc transcriptionally induce ODC expression [269]. Polyamine synthesis is correlated with cell growth and cancer, with a number of human and rodent cancers having elevated levels of polyamines and/or ODC activity [271]. High levels of polyamines enhance cell proliferation, reduce apoptosis, induce angiogenesis and the expression of genes involved in invasion and metastasis [271].

ODC activity is elevated in mouse skin papillomas compared to normal skin, with an even further increase in activity being found in carcinomas [270]. Topical application of diverse skin tumor promoters, including phorbol esters, teleocidin, okadaic acid, anthrones and calyculin A as well as UV irradiation and skin wounding, which also acts as a skin tumor promoter, all induce large increases in epidermal ODC expression and activity, albeit with different kinetics [270,272,273,274,275,276]. However, since other potent skin tumor promoters such as palytoxin do not induce ODC activity [277] and since TPA induces ODC activity to a similar level in skin tumor-resistant C57BL as in sensitive SSIN mice [278], ODC induction alone is not sufficient nor the only means to induce the epidermal hyperplasia related to skin tumor promotion.

However, elevated ODC activity in sensitive mice does promote both chemically- and UV-induced skin carcinogenesis. Treatment of mice with α-difluoromethylornithine (DFMO), an irreversible inhibitor of ODC, potently inhibits both chemical- and UV-induced mouse skin carcinogenesis [279,280]. Similarly, administration of DFMO in the drinking water to K14.HPV16 transgenic mice completely prevents the development of macroscopic and microscopic skin cancers induced by the expression of HPV16 [281]. Likewise, DFMO in the drinking water administered from birth to transgenic mice that overexpress the MAPK kinase upstream of ERK1/2, MEK, *via* a K14 promoter significantly delays and inhibits spontaneous tumor development [282]. Transgenic mice that overexpress ODC in multiple tissues *via* the mouse ODC promoter, in hair follicles *via* a K6 promoter or in the basal cells of the epidermis *via* a K5 promoter are more susceptible to skin tumorigenesis than wild-type mice [283,284]. A single dose of DMBA, without application of tumor promoters, is enough to induce skin tumor development in both the K6.ODC and K5.ODC mice, which indicates that overexpression of ODC is sufficient to promote tumorigenesis [284]. Similarly, when initiation is accomplished by expression of v-H-*ras* (Tg.AC transgenic mice), overexpression of ODC is able to promote spontaneous skin tumor development (Tg.AC/K6.ODC bigenic mice) [285]. Conversely, reduced expression of ODC in heterozygous ODC null mice significantly inhibits DMBA/TPA-induced skin tumorigenesis [286]. Likewise, transgenic mice that overexpress antizyme from either a K5 or a K6 promoter have reduced TPA induction of ODC activity and are resistant to DMBA/TPA skin tumorigenesis [287]. K5 or K6.antizyme transgenic mice also show delayed and reduced K14.MEK-driven skin tumor development [288]. Together all these results indicate that elevation of ODC activity is an important and critical mechanism in skin tumor promotion.

In addition to tumor promotion, ODC activity also plays a role in tumor maintenance. Tumor regression results when DFMO treatment is begun after skin tumors have developed. This has been shown with UV-induced tumors [279,289] and with spontaneous tumors from Tg.AC/K6.ODC bigenic mice [290], K14.MEK transgenic mice [282] and K14.HPV16 transgenic mice [281]. In Tg.AC/K6-ODC bigenic mice, inhibition of ODC activity induces apoptosis and reduces vascularization in DFMO-regressed tumors, but does not inhibit proliferation or reduce cyclin D1 expression [290]. This suggests that ODC activity/polyamines are activating cell survival and angiogenic pathways that are necessary for tumor maintenance rather than driving proliferation in these tumors.

In primary keratinocytes from K6.ODC transgenic mice, overexpression of ODC leads to activation of Akt/mTOR and Rho/Rac signaling pathways [291]. Chromatin modifications and altered gene expression also result from overexpression of ODC. The epidermis and skin tumors of Tg.AC/K6.ODC bigenic mice have elevated histone acetyltransferase (HAT) activity, specifically acetylating Lys^12^ of histone H4 [292]. The HAT responsible is Tip60 and elevated Tip60 protein, but not mRNA, is also seen in the skin of K6.ODC monogenic mice [293]. Increased Tip60 levels are found in association with a subset of transcription factors, including E2F1, resulting in binding to the promoters and up-regulation of expression of E2F1 target genes [293]. Thus, high levels of ODC and dysregulated polyamine synthesis promote tumorigenesis and tumor maintenance by affecting pro-survival and proliferative pathways and the transcriptional program of cells.

## 6. Conclusions

In conclusion, it is clear that activation of multiple molecular mechanisms and signaling pathways can be utilized to promote skin tumorigenesis. Induction of a sustained proliferation and hyperplasia is an effect of most tumor promoters. Proliferation can be induced directly by tumor promoter activation of mitotic pathways, with repeated applications of the promoter being necessary to maintain proliferation and hyperplasia. Alternatively, chronic tissue injury induced in response to chronic inflammation and generation of cytoxic ROS leads to a sustained regenerative proliferation. Proliferation is necessary to fix the initiating mutation and to drive clonal expansion of initiated cells that have altered responses to growth promoting or inhibitory signals. The EGFR signaling pathway is often activated by tumor promoters, either by induction of EGFR ligand expression, processing and release of membrane-bound EGFR ligands or by ligand-independent activation mediated by PKC/MAPK or c-Src phosphorylation, or by inactivation of receptor tyrosine phosphatase. EGFR signaling is a major mitogenic pathway for keratinocytes.

Another common feature of tumor promotion is chronic inflammation. Acute inflammation is an important physiological response to microorganisms, wounding and some toxic chemicals, but it is limited and the inflammation resolves after the trigger is eliminated, healed and/or detoxified. However, a situation with sustained, chronic inflammation promotes carcinogenesis. Tumor promoter activation of the NF-κB signaling pathway regulates the expression of many pro-inflammatory molecules that recruit the influx of inflammatory cells into the dermis. Infiltrating inflammatory cells then secrete cytokines and growth factors that induce proliferation of the adjacent keratinocytes. In addition, inflammatory cell-derived ROS creates a pro-oxidant environment, thereby altering signaling pathways and gene expression. Finally, persistent inflammation leads to tissue damage and cell turnover.

Oxidative stress generated not only from inflammatory cells, but also induced within keratinocytes by tumor promoters, is also frequently seen in the skin tumor promotion model. ROS generated within keratinocytes can form oxidative DNA adducts and lead to DNA mutations, which can initiate tumorigenesis, as well as promote tumorigenesis by altering the expression or function of key signaling molecules and contribute to increasing genomic instability.

Another common theme is the sustained activation of signaling pathways. To maintain persistent proliferation and inflammation needed for tumor promotion, signaling pathways have to be continuously activated. Most of the signaling pathways mentioned in this review are normally only transiently activated and have mechanisms, such as internalization and degradation of membrane receptors, to keep signaling limited. Tumor promotion results from continued, persistent activation of multiple signaling pathways.

Because many human cancers develop through a multi-step process as in the mouse skin model, elucidating the molecular mechanisms that promote skin tumor development should lead to better approaches to human cancer prevention, intervention and therapy. 
